# The Ferroelectric Domain Structures Induced by Electron Beam Scanning in Lithium Niobate

**DOI:** 10.1155/2018/7809826

**Published:** 2018-02-01

**Authors:** Evgeny Vlasov, Dmitry Chezganov, Maria Chuvakova, Vladimir Ya. Shur

**Affiliations:** ^1^School of Natural Sciences and Mathematics, Ural Federal University, Ekaterinburg, Russia; ^2^Labfer Ltd., Ekaterinburg, Russia

## Abstract

Ferroelectric domain structure has been formed under the action of electron beam scanning in congruent lithium niobate single crystal covered by surface dielectric layer. The obtained types of the domain patterns have been considered as subsequent stages of domain structure evolution. The dependence on irradiated charge density of domain density, length, and period of domain rays and stripe domain width was used for characterization of the domain structure evolution. The threshold irradiated charge density necessary for the formation of solid stripe domain has been revealed. All obtained results have been discussed in terms of kinetic approach based on the analogy between domain structure evolution and first-order phase transition.

## 1. Introduction

Nowadays, one of the most important goals of the nonlinear optics is expansion of the frequency range of the generated coherent radiation. It has become achievable due to development of the domain engineering methods in nonlinear optical ferroelectric crystals [1]. The periodically poled (PP) crystals allow producing the devices for quasi-phase matched frequency conversion by second harmonic generation and optical parametric oscillation [2–4]. The lithium niobate (LiNbO_3_, LN) is one of the most attractive materials for these applications due to its high nonlinear and electrooptical coefficients [5].

The traditional technique of domain engineering based on application of electric field by stripe electrodes faces such problems as domain broadening and spontaneous backswitching, which hamper achieving the submicron periods [6, 7]. These disadvantages stimulate development of alternative techniques of domain writing by charged particle irradiation [8–17].

The switching by an electron beam is very promising, because the size of the electron beam is easily scaled down to several nanometers, which allows overcoming the limitations of the traditional method. The method modification by coating of the irradiated surface by buffer dielectric layer has been successfully used to create PPLN with high homogeneity and quality [18–20]. The deposition of the artificial dielectric layer leads to (i) localization of the injected charge, (ii) decreasing the “charging effect,” (iii) reducing the threshold irradiated charge density, and (iv) decreasing the accelerating voltage [20]. We have demonstrated recently the PPLN with periods from 6 to 16 *μ*m produced by e-beam for second harmonic generation with high conversion efficiency in the bulk crystals of MgO-doped LN (MgOLN) [21] and soft proton exchanged channel waveguides in LN [22].

It is well known that existence of the intrinsic or artificial surface dielectric layer leads to qualitative change of the mechanisms of the domain structure evolution, appearance of the nanodomain ensembles, and lack of the domain wall shape stability [23, 24]. These effects were obtained for e-beam poling also, but, to our knowledge, they have never been studied [18]. The further development of the periodical poling by e-beam and creation of submicron patterns needs deeper understanding of the first stages of domain structure evolution including formation of the self-assembled nanodomains [25].

This paper is devoted to investigation and analysis of domain structure formation and domain shapes created by e-beam irradiation in the bulk congruent lithium niobate (CLN) crystals covered by artificial dielectric layer.

## 2. Materials and Methods

We have studied the 0.5 mm thick *Z*-cut plates of CLN single crystals (Crystal Tech., USA). The irradiated *Z*− polar surface was covered by 2.5 *μ*m thick AZ nLOF 2020 (Microchemicals GmbH, Germany) photoresist layer by spin-coating method. The solid copper electrode was deposited by the magnetron sputtering at the opposite polar surface and grounded during irradiation.

The samples were irradiated in the vacuum chamber of the Auriga Crossbeam workstation (Carl Zeiss, Germany) equipped with electron beam lithography system Elphy Multibeam (Raith GmbH, Germany). The lithography system was used for precise control of the exposure parameters and beam movement. The irradiated patterns were created using Raith Nanosuite software and represented a grating of stripes oriented along *Y*-crystallographic axis with period of 20 *μ*m and width of 750 nm. The stripe exposure was performed by transversal meander-scan covering of the desired area. The samples were irradiated at the fixed accelerating voltage of 14 kV and the beam current of about 1 nA. The charge density *D* has been used for characterization of the irradiation condition.

(1) $$\begin{array}{c} D=\frac{I\times t}{A}, \end{array}$$

where *I* is electron beam current, *t* is exposure time, and *A* is irradiated area.

The static domain structures were revealed by selective chemical etching in pure HF for 90 s at the room temperature after removal of the dielectric layer and electrode [26, 27]. The surface relief was visualized by scanning electron microscopy using secondary electron detection mode with resolution down to 2 nm at the primary electron energy of 3 keV [28].

## 3. Results and Discussion

**Figure 1:**
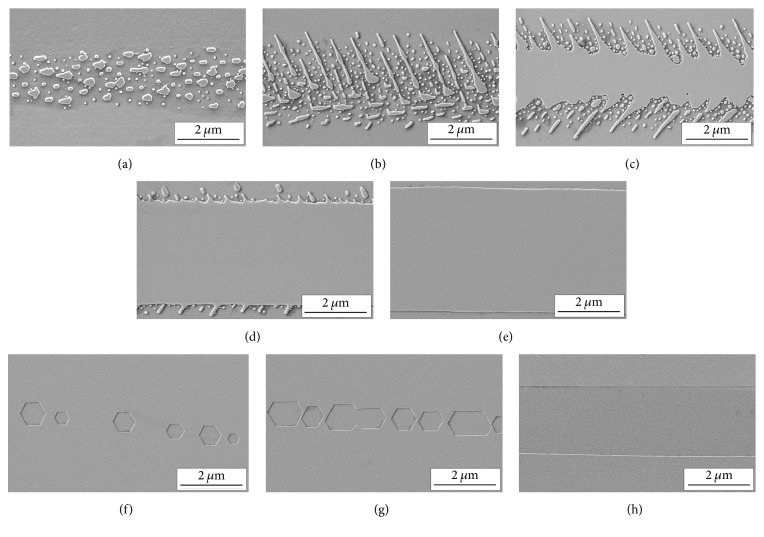
SEM images of domains created by e-beam at different charge densities: (a) 1 mC/cm^2^; (b), (f) 2 mC/cm^2^; (c), (g) 26 mC/cm^2^; (d) 32 mC/cm^2^; (e), (h) 56 mC/cm^2^. (a)–(e) *Z*−; (f)–(h) *Z*+. Stages of domain growth: (a) discrete switching, (b) domain ray growth, (c) domain merge, (d) formation of stripe domains, and (e) widening of stripe domain.

**Figure 2:**
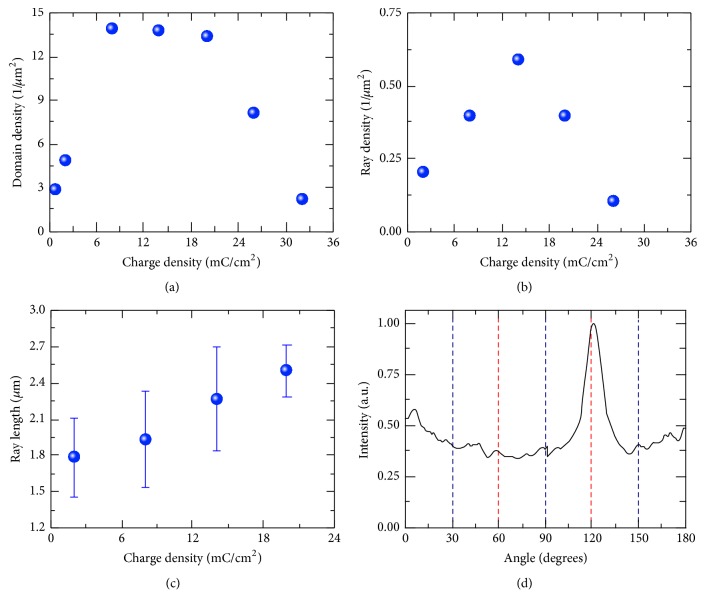
The charge density dependence of (a) nanodomain density, (b) ray density, and (c) ray length. (d) Angular distribution of ray directions: red lines correspond to *Y*-crystallographic axis and blue lines to *X*-crystallographic axis.

**Figure 3:**
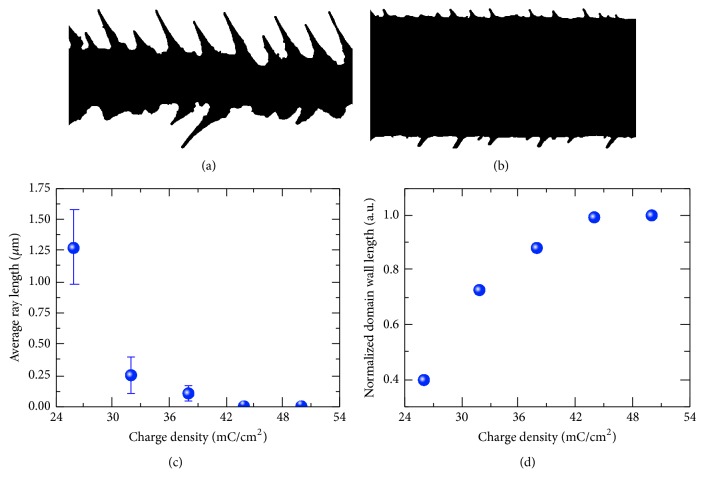
(a), (b) Binary images of solid domains with jagged domain walls for charge density of (a) 26 mC/cm^2^ and (b) 32 mC/cm^2^. The charge density dependence of (c) the average ray length and (d) the normalized domain wall length.

**Figure 4:**
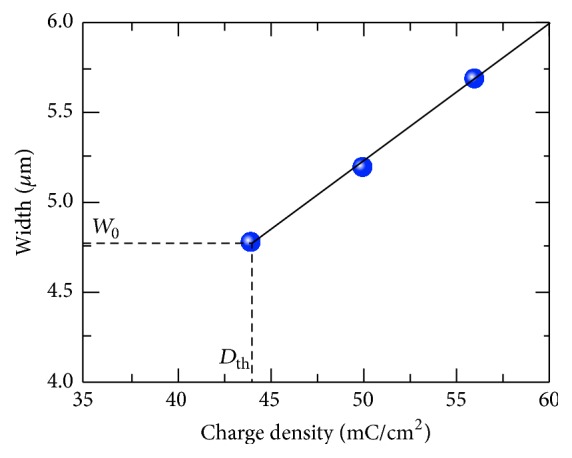
The charge density dependence of the stripe domain width.

We have investigated the charge density dependence of the domain shape obtained after the stripe irradiation for *D* range from 1 to 56 mC/cm^2^. Four types of domain structures were distinguished: (1) isolated nanodomains (Figure 1(a)), (2) isolated domain rays oriented along *Y*+ crystallographic direction (Figure 1(b)), (3) solid domains with jagged domain walls (“fish-bone” structure [25]) (Figures 1(c) and 1(d)), and (4) continuous solid stripe domains (Figure 1(e)).

The domains grew through the crystal and appeared at the opposite polar surface as quasi-periodic chains of isolated hexagonal microdomains (Figure 1(f)), which merged to the “dashed” structure (Figure 1(g)) for middle *D* values. Continuous solid stripe domains appeared for *D* above 56 mC/cm^2^ (Figure 1(h)). The similar behavior was obtained as a result of e-beam irradiation in MgOLN [29]. The classical hexagonal domain shape at *Z*+ polar surface can be explained by effective screening conditions produced by the metal electrode grounded during irradiation [24].

It should be noted that in our experiments the irradiation times (about few milliseconds) have been several orders of magnitude shorter than the switching times (close to one second). Thus, the irradiation with various charge densities can be considered as an analog of the switching by field pulses of various amplitudes and durations. Therefore, the different types of domain structure have been considered as different stages of domain structure evolution. This approach allowed distinguishing five subsequent stages of domain growth: (1) discrete switching (appearance of isolated nanodomains) (Figure 1(a)), (2) domain ray growth (Figure 1(b)), (3) domain merge leading to formation of fish-bone structure (Figure 1(c)), (4) formation of stripe domains (Figure 1(d)), and (5) widening of stripe domain by sideways domain wall motion (Figure 1(e)).

### 3.1. Discrete Switching and Domain Ray Growth

The domain density, as well as length and period of domain rays, was used for quantitative characterization of domain evolution at the first two stages with increasing charge density. The *D* dependence of isolated domain density demonstrates three regions with different behaviors (Figure 2(a)). The sharp rise at low *D* (below 8 mC/cm^2^) corresponds to intensive nucleation (Figures 1(a) and 1(b)). The slow decrease of domain density was attributed to rays broadening due to domain merging. The final rapid fall of nanodomain density is caused by formation and broadening of solid domain and subsequent sideways wall motion by merging with existing nanodomains (Figures 1(c) and 1(d)). The *D* dependence of ray density demonstrated increase and decrease regions (Figure 2(b)). The increase of the ray number obtained for *D* below 14 mC/cm^2^ is due to appearance of new rays, while the subsequent decrease is due to ray merging.

The short and long domain rays have been separated. The short rays appeared between long ones randomly. The average period of long domain rays (about 1 *μ*m) keeps constant with *D* increase. This fact can be attributed to correlated nucleation effect (appearance of the local field maximum in front of the moving domain wall) [30]. It has been shown that the field maximum located at the distance from the domain wall equal to the dielectric layer thickness leads to the formation of nanodomains in front of the moving wall [24, 25]. The decrease of the electric field in the vicinity of isolated domain leads to domain formation at the given distance from existing domain wall. It is known that the new maximum of local electric field appears at the same distance from the first domain, which resulted in formation of the second one. Thus, the formation of the periodic long-ray structure can be caused by existence of the effective dielectric layer with thickness equal to the distance between the edge of the space charge region and LN surface (about 1 *μ*m) [21].

The linear increase of the average ray length with *D* increase (Figure 2(c)) is due to increase of electric field generated by injected charge.

The Fourier analysis of the domain patterns allows revealing angular distribution of the ray directions. The prevalent ray orientation along the *Y*-crystallographic axis is caused by anisotropy of the bulk screening [24, 25]. It should be noted that the interaction with the earlier appearing domains led to prevalence of the ray growth in “free” direction (Figure 2(d)).

### 3.2. Domain Merging and Stripe Domain Formation

We have used charge density dependence of the average ray length and the normalized domain wall length obtained by analysis of the binary domain images for characterization of the stripe domain formation (Figure 3). The average ray length decreased with *D* increase up to 44 mC/cm^2^ and disappeared for larger *D* corresponding to formation of the flat domain wall (Figure 3(c)). The normalized domain wall length has been calculated as *L* = *l*_fd_/*l*_*d*_, where *l*_*d*_ is the domain wall length and *l*_fd_ is the length of the flat boundary (Figure 3(d)). Both approaches have been used for measuring of the threshold *D* necessary for formation of solid stripe domain.

### 3.3. Widening of Stripe Domain

We have measured the charge density dependence of domain width as a characteristic of sideways domain wall motion. The similar linear dependence (Figure 4) has been obtained earlier in MgOLN and soft proton exchanged congruent LN [22, 29]. The dependence can be attributed to linear increase of the surface potential and external electric field with irradiation time in the studied interval of the charge density. It is necessary to bear in mind that the irradiated charge is used partially for compensation of the depolarization field (screening). Thus the switched area is proportional to excess of the charge density over its threshold value. The obtained dependence was fitted by

(2) $$\begin{array}{c} W=W_{0}+C\left(\;D-D_{\text{th}}\;\right), \end{array}$$

where *W* is the stripe domain width, *W*_0_ is the minimal width, *D*_th_ is the threshold charge density necessary for formation of stripe domain, and *C* is constant. The parameters of linear fitting by (2) are presented in Table 1.

## 4. Conclusions

The domain structure formation induced by electron beam irradiation has been studied in congruent lithium niobate single crystal covered by surface dielectric layer. The different types of domain structures have been revealed at the irradiated surface depending on irradiated charge density. The exposure with different charge densities has been considered as an analog of the switching. Thus, the irradiation with various charge densities can be considered as an analog of the switching by field pulses of various amplitude and duration, and the different types of domain structure have been considered as different stages of domain structure evolution.

The processes of discrete switching and domain ray growth have been characterized by domain density, as well as length and period of domain rays as a function of charge density. The prevalent orientation of the domain rays along *Y*-crystallographic axis has been attributed to screening anisotropy. The domain merging leading to formation of stripe domain has been considered. The threshold value of irradiated charge density necessary for formation of solid stripe domain has been revealed. The linear dependence of domain width on irradiated charge density correlates with results obtained recently in other materials. All obtained results have been explained in terms of kinetic approach based on analogy of domain growth with first-order phase transition. The ray formation at the irradiated surface was attributed to ineffective screening of depolarization field due to existence of artificial dielectric layer.

## Figures and Tables

**Table 1:** Parameters of linear fitting of charge density dependence of domain width.

	Value	Standard error
*W*_0_, *μ*m	4.77	0.17
*D*_th_, mC/cm^2^	44.0	1.4
*C*, cm^3^/C	0.076	0.003
